# Identification of Cell Markers and Their Expression Patterns in Skin Based on Single-Cell RNA-Sequencing Profiles

**DOI:** 10.3390/life12040550

**Published:** 2022-04-07

**Authors:** Xianchao Zhou, Shijian Ding, Deling Wang, Lei Chen, Kaiyan Feng, Tao Huang, Zhandong Li, Yudong Cai

**Affiliations:** 1 School of Life Sciences, Shanghai University, Shanghai 200444, China; zhouxch1@shanghaitech.edu.cn (X.Z.); dingshijian@shu.edu.cn (S.D.); 2 Center for Single-Cell Omics, School of Public Health, Shanghai Jiao Tong University School of Medicine, Shanghai 200025, China; 3 State Key Laboratory of Oncology in South China, Collaborative Innovation Center for Cancer Medicine, Department of Medical Imaging, Sun Yat-sen University Cancer Center, Guangzhou 510060, China; wangdl@sysucc.org.cn; 4 College of Information Engineering, Shanghai Maritime University, Shanghai 201306, China; lchen@shmtu.edu.cn; 5 Department of Computer Science, Guangdong AIB Polytechnic College, Guangzhou 510507, China; kyfeng@gdaib.edu.cn; 6 Bio-Med Big Data Center, CAS Key Laboratory of Computational Biology, Shanghai Institute of Nutrition and Health, University of Chinese Academy of Sciences, Chinese Academy of Sciences, Shanghai 200031, China; 7 CAS Key Laboratory of Tissue Microenvironment and Tumor, Shanghai Institute of Nutrition and Health, University of Chinese Academy of Sciences, Chinese Academy of Sciences, Shanghai 200031, China; 8 College of Food Engineering, Jilin Engineering Normal University, Changchun 130052, China

**Keywords:** skin disease, cell marker, expression pattern, feature selection, classification algorithm, rule learning

## Abstract

Atopic dermatitis and psoriasis are members of a family of inflammatory skin disorders. Cellular immune responses in skin tissues contribute to the development of these diseases. However, their underlying immune mechanisms remain to be fully elucidated. We developed a computational pipeline for analyzing the single-cell RNA-sequencing profiles of the Human Cell Atlas skin dataset to investigate the pathological mechanisms of skin diseases. First, we applied the maximum relevance criterion and the Boruta feature selection method to exclude irrelevant gene features from the single-cell gene expression profiles of inflammatory skin disease samples and healthy controls. The retained gene features were ranked by using the Monte Carlo feature selection method on the basis of their importance, and a feature list was compiled. This list was then introduced into the incremental feature selection method that combined the decision tree and random forest algorithms to extract important cell markers and thus build excellent classifiers and decision rules. These cell markers and their expression patterns have been analyzed and validated in recent studies and are potential therapeutic and diagnostic targets for skin diseases because their expression affects the pathogenesis of inflammatory skin diseases.

## 1. Introduction

**Figure 1:**
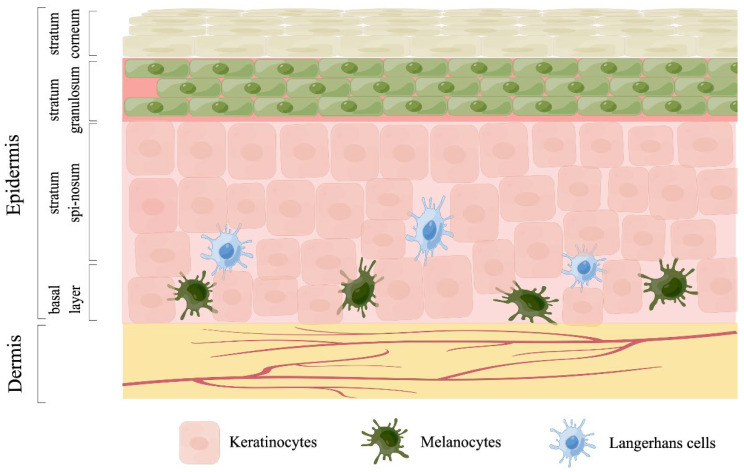
Structure of the skin. The skin is mainly divided into epidermis and dermis. The epidermis layer can be further divided into stratum corneum, stratum granulosum, stratum spinosum, and basal layer, and is mainly composed of keratinocytes, melanocytes, and Langerhans cells. The dermis is much thicker than the epidermis and is mainly composed of collagen and elastin, which make the skin elastic and stretchable. This figure was plotted by Figdraw (www.figdraw.com, accessed on 13 March 2022).

The skin is the largest and most extensive organ of the human body, accounting for approximately 15% of the total body weight of adult humans [1]. It serves as a barrier between the body and the external environment and has diverse functions, such as body protection, waste excretion, body temperature regulation, and sensory perception [2]. The skin can be divided into the epidermis and dermis, from the outside to the inside. The epidermis can be divided into the stratum corneum, stratum granulosum, stratum spinosum, and basal layer, from top to bottom, as illustrated in Figure 1. It is mainly composed of keratinocytes, melanocytes, and Langerhans cells. Among these cells, keratinocytes are predominant. They are produced in the basal layer and continuously migrate outward during differentiation and maturation to form the stratum spinosum, stratum granulosum, and stratum corneum. Melanocytes and Langerhans cells are specific dendritic cells that are distributed near the basal layer and are responsible for pigment synthesis and antigen presentation, respectively [3]. The dermis is mainly composed of fibroblasts and is rich in blood vessels, lymphatic vessels, and nerve endings. It can be divided into two regions: the papillary dermis and the deeper reticular dermis. The deeper reticular dermis, which is largely acellular and rich in extracellular matrix (ECM) components, can confer physical strength, flexibility, and support to the skin [4,5].

Atopic dermatitis (AD) and psoriasis are inflammatory skin diseases that are believed to be the result of the interaction of immune cells and keratinocytes. AD is a multifactorial and complex disease that is characterized by pruritic, erythematous, and scaly skin lesions; its pathogenic trigger has been proposed to comprise environmental and genetic factors and immune response dysregulation [6]. Previous studies have shown that a variety of immune cells in patients with AD are abnormal. For example, in patients with AD, Th2, eosinophils, and IgE production are increased and interleukin is upregulated [7,8]. Psoriasis is another immune-related disease that is characterized by accelerated epidermal proliferation, cellular influx, and inflammatory mediators [9,10]. A variety of interleukins that are produced by inflammatory myeloid dendritic cells can activate multiple types of T cells. These T cells produce abundant psoriatic cytokines, which further affect keratinocytes and promote psoriasis [11,12].

Given that AD and psoriasis are closely related to immune disorders, understanding the interaction between immune cells and keratinocytes is extremely important. Advances in single-cell sequencing have provided us with precise tools for the comprehensive analysis of these diseases. By using single-cell sequencing, researchers can clearly analyze each type of cell in an inflammatory skin disease sample and characterize the expression patterns of different cell populations and the relationship between them. Previous single-cell studies have found some rare subpopulations, including inflammatory fibroblasts, that are unique to patients with AD and that AD lesions are characterized by enlarged type 2 T cells and inflammatory DC. However, the interaction of cells with inflammatory fibroblasts and immune cells in damaged skin is largely unknown and needs to be further studied [13]. Another single-cell study identified the molecular characteristics of multiple monocyte-derived cells in patients with inflammatory skin diseases. Research on the regulatory, repair, or anti-inflammatory functions of these cell types may help develop new treatment strategies for inflammatory skin diseases [14]. One study also utilized single-cell sequencing technology to map the immune landscape of the synovium in psoriatic arthritis and observe changes in CD8 T cell clones; this study may provide a reference for studying the driving antigens of psoriatic arthritis [15]. A recent paper published in *Science* determined the differences in immune cell composition in two inflammatory skin diseases (AD and psoriasis) by comparing fetal and healthy adult skin and thus provided a road map for the pathological processes of inflammatory skin diseases [16]. The present study further investigated the data reported in the previous study.

In contrast to previous single-cell studies on inflammatory skin diseases, our study compared the gene expression levels of different cell types in inflammatory skin disease samples and healthy controls by using several machine learning methods, which constituted a computational pipeline. Original gene features in the expression profiles were filtered one by one by the maximum relevance criterion and Boruta feature selection method [17]. Irrelevant gene features were excluded, and the remaining ones were further analyzed by Monte Carlo feature selection (MCFS) [18], resulting in a feature list. Such a list was fed into the incremental feature selection (IFS) method [19], incorporating random forest (RF) [20] and decision tree (DT) [21] as classification algorithms. Through such a procedure, we obtained essential gene features and decision rules that may be crucial for the classification of 114 cell types. The genes can be latent cell markers, and rules can indicate different expression patterns in different cell types. Furthermore, an efficient RF classifier was built, which can be a useful tool to identify cell types. The findings reported in this analysis may provide novel insights into the pathogenesis of inflammatory skin disease, as well as a reference for the development of new therapies.

## 2. Materials and Methods

### 2.1. Single-Cell RNA-Sequencing Profiles of Skin Samples

We downloaded the single-cell expression profiles of 451,280 cells of 114 cell types in inflammatory skin disease samples and healthy controls from https://developmentcellatlas.ncl.ac.uk/datasets/hca_skin_portal (accessed on 15 January 2021) [16]. The sample sizes of each cell type are presented in Table S1. The expression levels of 33,538 genes were assessed in a previous study [16]. Our purpose is to distinguish cell types at the single-cell level.

### 2.2. Feature Selection

Analyzing all gene features was difficult because each cell sample had 33,538 features in its original single-cell profiles. The feature selection method is an excellent way to extract essential features from such large profiles. Here, we adopted several feature selection methods or criteria.

#### 2.2.1. Feature Exclusion Based on the Maximum Relevance Criterion

We first applied the maximum relevance criterion to exclude the most unrelated features.

The definition of the maximum relevance criterion suggests that predictive features should exhibit a high degree of correlation with the label variable. The correlation between features and the label variable was computed by using mutual information (MI), which is defined as follows:

(1) $$I\left(x,y\right)=\int \int p\left(x,y\right)log\frac{p\left(x,y\right)}{p\left(x\right)p\left(y\right)}dxdy,$$

where p(x) and p(y) are the marginal probability densities of *x* and *y*, respectively, and p(x,y) is the joint probability density of *x* and *y*. In the present study, to compute the MI value of each gene feature, we adopted its codes integrated into the mRMR program, which can be downloaded from http://penglab.janelia.org/proj/mRMR/ (accessed on 2 May 2018). A threshold of 0.001 was used to exclude the most unrelated features. Retained features would be analyzed by using the following feature selection methods.

#### 2.2.2. Boruta Feature Filtering

The remaining features were further analyzed by Boruta [17], which is a wrapper feature selection method that is based on the RF algorithm. It assesses the importance of features by comparing the features with shuffled features. First, it attempts to add randomness to a specific dataset by creating the shuffled duplicates of all features, which are called shadow features. Then, an RF classifier is used to train the generated dataset and evaluate the importance of each feature in accordance with the evaluation metrics. High performance indicates high importance. In each iteration, it checks whether the importance of a real feature is higher than the values of its shadow features and continuously removes features that are considered very nonimportant. Finally, the Boruta stops running when all features are confirmed or denied or when a specified limit of the RF operation is reached. In this study, we applied the Boruta program written in Python language, which was retrieved from https://github.com/scikit-learn-contrib/boruta_py (accessed on 14 September 2020). Parameters in this program were set to default values. The selected features would be investigated by the following MCFS method.

#### 2.2.3. Monte Carlo Feature Selection

For the gene features selected by Boruta, they were finally analyzed by the MCFS method. This is a powerful feature selection method that evaluates the relative importance (RI) of features on the basis of a DT algorithm [18]. First, it randomly selects *s* subsets of *m* features from all *d* features, where *m* << *d*. Then, *t* trees can be trained on randomly selected samples that are represented by features in each subset, and the performance of these trees is assessed. With the above procedures, s×t trees are established and evaluated. The RI score of one feature *f* is calculated as follows, to assess the importance of features in these trees:

(2) $$RI_{f}=\sum_{\tau =1}^{s\times t}{\left(wAcc\right)}^{u}IG\left(n_{f}\left(\tau \right)\right){\left(\frac{\mathrm{no}.\mathrm{in}\;n_{f}\left(\tau \right)}{\mathrm{no}.\mathrm{in}\;\tau }\right)}^{v},$$

where wAcc and $IG\left(n_{f}\left(\tau \right)\right)$ are the weighted accuracy of DT τ and the information gain of node $n_{f}\left(\tau \right)$, respectively; $\mathrm{no}.\mathrm{in}\;n_{f}\left(\tau \right)$ and no.in τ refer to the number of samples of $n_{f}\left(\tau \right)\;$and τ, respectively; u and v are the weighting coefficients with a default setting value of 1. Finally, a ranked feature list *F* is produced on the basis of the decreasing order of the evaluated RI scores.

The MCFS program utilized in this analysis was obtained from http://www.ipipan.eu/staff/m.draminski/mcfs.html (accessed on 4 June 2019), and the default parameters were used.

### 2.3. Incremental Feature Selection

Although the features were ranked by using the MCFS method, the best number of features that can be used to classify different cell types could not be determined. We applied the IFS method [19] to identify the optimal number of features and construct the best classifier. At the same time, features used in the best classifier were obtained, which can be important for classifying cell types. Specifically, based on the feature list (e.g., the list *F* generated by MCFS), a series of feature subsets with a step size was constructed. For example, when the step size was set to five, the first subset included the top five features in the list, and the second subset was composed of the top 10 (5×2) features. Then, for each feature subset, a classifier with a given classification algorithm (e.g., DT [21] or RF [20]) was trained on the samples that were represented by features in this subset. Its performance was evaluated via 10-fold cross-validation [22]. The classifier providing the best performance was obtained, which was called the optimal classifier. Additionally, features used in such an optimal classifier were termed optimal features.

### 2.4. Synthetic Minority Oversampling Technique

As listed in Table S1, the largest cell type contained more than 35,000 samples, whereas the smallest cell type only contained 111 samples. Thus, the dataset was class-unbalanced. The classifier directly built on such a dataset may produce bias. To overcome this problem, the synthetic minority oversampling technique (SMOTE) [23] method was employed, which can amplify samples in minority classes. Specifically, for a randomly selected sample *x* in a minority class, the distance from it to all samples in this minority class is measured by using the Euclidean distance, and its *k*-nearest neighbors are obtained. One neighbor, e.g., *y*, is randomly selected. A new sample *z* is produced, which is defined as the linear combination of *x* and *y*. Such a new sample *z* is also put into the minority class. The above procedure stops until the minority class has the same number of samples as the majority class. Finally, all classes contain the same number of samples. In the present study, the SMOTE program that was obtained from https://github.com/scikit-learn-contrib/imbalanced-learn (accessed on 24 March 2020), was applied to balance the data, and the default parameters were used. It is necessary to note that SMOTE was only used in evaluating the performance of classifiers in the IFS method.

### 2.5. Classification Algorithms

To perform the IFS method, one classification algorithm is necessary. This study selected RF [20] and DT [21]. A brief introduction to these methods is provided in what follows.

#### 2.5.1. RF

RF [20] is an ensemble learning method that trains and predicts a sample by using multiple trees. Each tree is constructed based on samples randomly selected from the original dataset. Then, features are randomly selected and used to grow the tree at each node. When using RF for classification, the prediction results are outputted by the majority voting of all trees. Although DT is a weak algorithm, RF is generally much more powerful. Several studies adopted it to build efficient classifiers [24,25,26,27,28,29,30]. In the present study, the RF program was performed by using the scikit-learn (https://scikit-learn.org/stable/, accessed on 3 November 2020) module [31], and the default parameters were used to construct classifiers.

#### 2.5.2. DT

Although we can build an efficient classifier using RF, its underlying principle is difficult to understand, preventing us from uncovering essential differences in various cell types. Accordingly, we also employed DT [21] in this study, which is a white-box model that can yield interpretable decision rules. DT is usually developed on the basis of the IF–THEN principle, starting with a single node that can branch into possible results. Each outcome leads to other nodes, which, in turn, branch into other possibilities, thus yielding a tree-like structure. From this tree, several rules can be obtained, each of which contains a condition and one result. In this study, the condition of one rule always involved several gene features, indicating a special expression pattern. The result of the condition was one cell type, suggesting the special expression pattern was an essential marker for this cell type. Further investigation of obtained rules can improve our comprehension of different cell types. Such merit of DT induces its wide applications in the biomedical field [32,33,34]. In this study, the scikit-learn (https://scikit-learn.org/stable/, accessed on 3 November 2020) module was applied with default parameters to build the DT.

### 2.6. Performance Measurement

The Matthews correlation coefficient (MCC) [35,36,37,38] was used as the evaluation metric to quantify the performance of all constructed classifiers. In multiple classification tasks, the MCC is defined as follows:

(3) $$\mathrm{MCC}=\frac{cov\left(x,y\right)}{\sqrt{cov\left(x,x\right)cov\left(y,y\right)}},$$

where *x* and *y* represent the binary matrixes of the true and predicted labels, respectively; and cov( ) denotes the covariance function between two factors, such as *x* and *y*. The value of MCC is in the range of −1 to 1, and a high MCC value indicates the strong performance of the classifier.

In addition to MCC, some other measurements were also calculated to fully evaluate the performance of all classifiers, including overall accuracy (ACC) and individual accuracy on each cell type. ACC was defined as the proportion of correctly predicted cell samples, and individual accuracy on one cell type was the ratio of the number of correctly predicted cell samples in this type and the total number of cell samples in such type. MCC was selected as the key measurement, whereas others were provided for reference.

## 3. Results and Discussion

**Figure 2:**
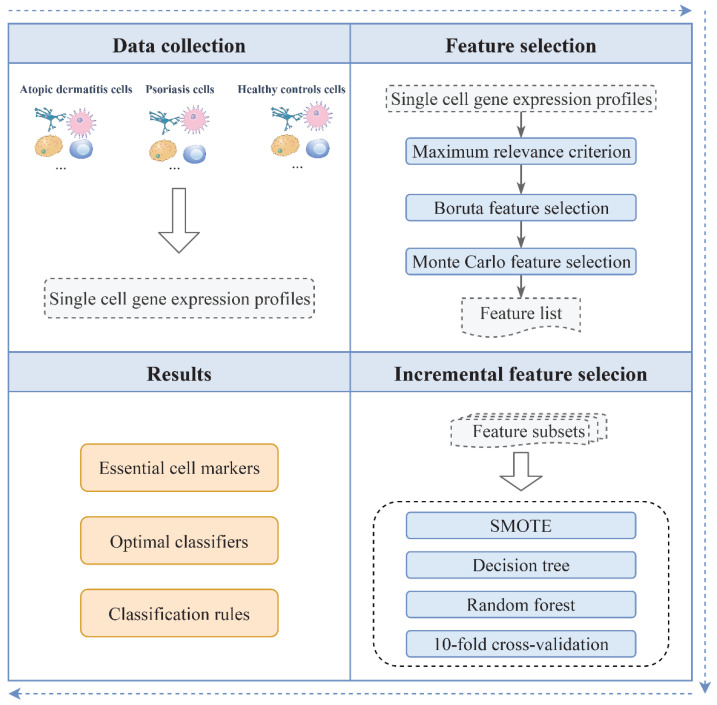
Whole computational pipeline of this study. First, the features in single-cell gene expression profiles of inflammatory skin disease were analyzed by using the maximum relevance criterion, Boruta feature selection, and MCFS methods, to obtain a feature list. This feature list was then fed into the IFS method with classification algorithms to extract important biomarkers and establish the optimal classifiers and classification rules.

In the present study, we proposed a computational pipeline, which contained several feature selection methods and classification algorithms, to identify essential biomarkers, build efficient classifiers, and extract decision rules. The whole procedure is illustrated in Figure 2.

### 3.1. Features Selected by Using the Maximum Relevance Criterion, Boruta, and MCFS Methods

We designed a workflow for feature selection to select important genes from the original single-cell expression profiles. First, we applied the MaxRel method to filter gene features on the basis of a cutoff score. A total of 18,023 features were selected in this step when the cutoff score was set to 0.001. Then, these features were subjected to the Boruta feature selection method to discard irrelevant features. Through the analysis, we removed 14,265 gene features and retained 3758 important gene features. These retained features are provided in Table S2. After the above process, the MCFS method was used to rank the remaining features in accordance with the evaluated RI scores. A ranked feature list, which is presented in Table S2, was obtained. The top five genes were CD74, HLA-DRA, HLA-DRB1, HLA-DPB1, and TYROBP. Through feature selection, we filtered and ranked 3758 important features from the original 33,538 features. This step facilitated the identification of the best genes, in addition to reducing the computational consumption of the next step.

### 3.2. Determination of the Optimal Features by the IFS Method

**Figure 3:**
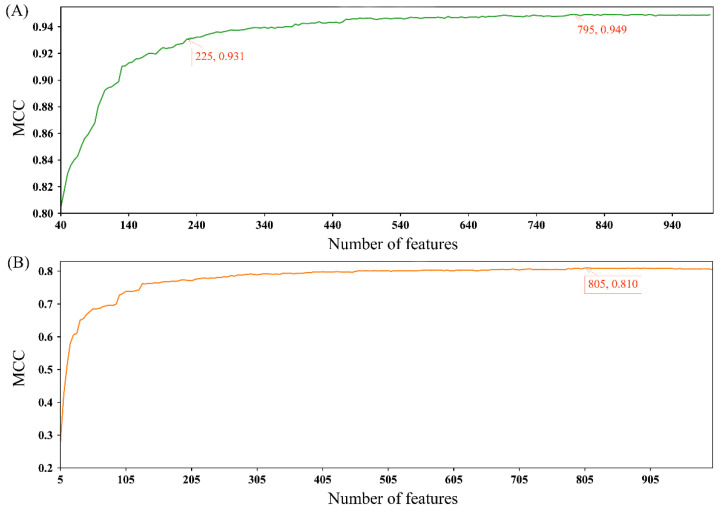
IFS curves of two classification algorithms: (**A**) IFS curve of the random forest; (**B**) IFS curve of the decision tree. The random forest provides the highest MCC of 0.949 when the top 795 features are used, whereas the decision tree yields the highest MCC of 0.810 when the top 805 features are adopted. The random forest with the top 225 features also produces a high MCC of 0.931.

**Figure 4:**
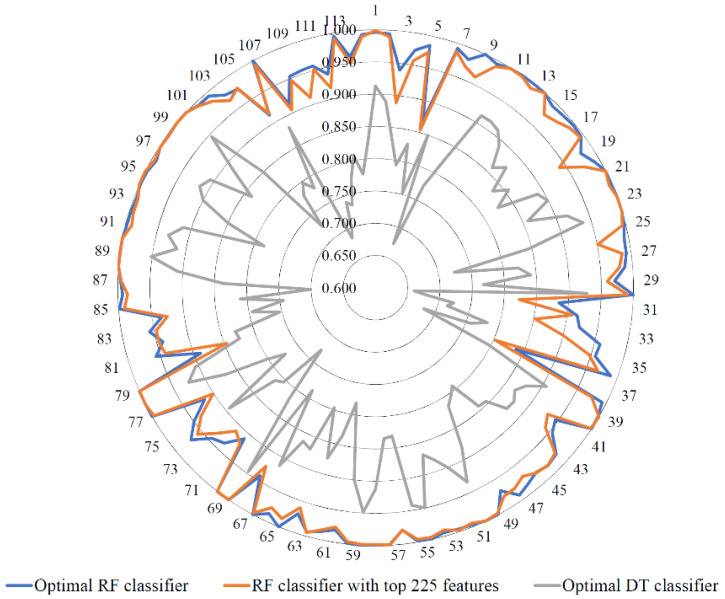
Performance levels of three key classifiers on 114 cell types. The optimal RF classifier and RF classifier with top 225 features provide almost equal performance, whereas the optimal DT classifier yields evident lower performance. Numbers 1–114 indicate the class indices of 114 cell types (see Table S1 for detailed data).

We still could not determine the number of features that can effectively classify cell types after the analysis with the MCFS method. Therefore, the IFS method with DT and RF algorithms was utilized to identify the optimal feature number. Several feature subsets were generated from the top 1000 features in the ranked feature list when the step size of IFS was 5. On each subset, an RF classifier was built and evaluated by using 10-fold cross-validation. The performance of these classifiers, including MCC, ACC, and individual accuracies on 114 cell types are presented in Table S3. For ease of viewing the performance of these RF classifiers, an IFS curve was plotted, with the number of features as the *x*-axis and the MCC as the *y*-axis (Figure 3A). Clearly, the highest MCC was 0.949 when the top 795 gene features were used. Accordingly, these features were optimal features for RF, and therefore, an optimal RF classifier was built on these features. The detailed predicted results of this classifier (confusion matrix) are provided in Table S4. The ACC of this classifier was 0.951, as listed in Table 1. The individual accuracies of all cell types are illustrated in Figure 4. In total, 25 cell types were perfectly predicted, and 110 (96.49%) types received individual accuracies higher than 0.900. All these indicated the superior performance of this optimal RF classifier. However, the efficiency of this classifier was not very high due to the number of features used. By carefully checking the performance of the RF classifier with fewer features, we found that when the top 225 features were used, the RF classifier can yield an MCC of 0.931. The confusion matrix of this classifier is provided in Table S4, indicating its high performance. The following 570 features can only improve MCC by 0.018. As listed in Table 1, the ACC of this RF classifier was 0.932, only 0.019 lower than that of the optimal RF classifier. Furthermore, the individual accuracies yielded by this classifier were also quite high, as shown in Figure 4. Cell samples in 16 types were perfectly predicted, and 108 individual accuracies were higher than 0.900, which indicates that this RF classifier provided almost equal performance to the optimal RF classifier. However, this classifier had higher efficiency than the optimal RF classifier because it needed much fewer gene features. The computation time of the 10-fold cross-validation on the optimal RF classifier was 14,722.01 s, whereas this time for the RF classifier with top 225 features was only 5300.73. This fact suggested that the RF classifier with top 225 features was much faster than the optimal RF classifier. It can be an efficient tool to determine cell types.

As mentioned in Section 2.5, RF can be helpful to build efficient classifiers. However, few insights can be obtained from these classifiers. Thus, we also employed DT. The same procedures were conducted for DT. Its performance on different top features is also presented in Table S3. An IFS curve was also plotted to show the performance of all DT classifiers, as illustrated in Figure 3B. It can be observed that the highest MCC was 0.810, which was obtained by using the top 805 features. Accordingly, an optimal DT classifier was built using these features. The detailed predicted results of this DT classifier are available in Table S4. The ACC of this DT classifier was 0.815, as listed in Table 1. Its performance on 114 cell types is shown in Figure 4. Evidently, this level of performance was much lower than that of the above-mentioned RF classifiers. However, the DT classifier has its own merit, not shared by the RF classifier. It can produce decision rules, which made the classification procedures completely open and also provided new insights to investigate the differences in various cell types of inflammatory skin disease. These rules are provided in Section 3.3.

In summary, the IFS method identified the best number of features for different classification algorithms, as well as established an efficient tool that can quickly classify different cell types of inflammatory skin disease.

### 3.3. Classification Rules Extracted by the Optimal DT Classifier

Although the optimal DT classifier had a weaker classification performance than that of the RF classifiers, it can provide interpretable rules that were useful for mining biological molecular mechanisms. The rules were generated by using the constructed optimal DT classifier. For further investigation, we extracted the top 1000 rules, which are listed in Table S5. These rules are stated in Section 3.6.

### 3.4. Computation Time vs. MCC

**Figure 5:**
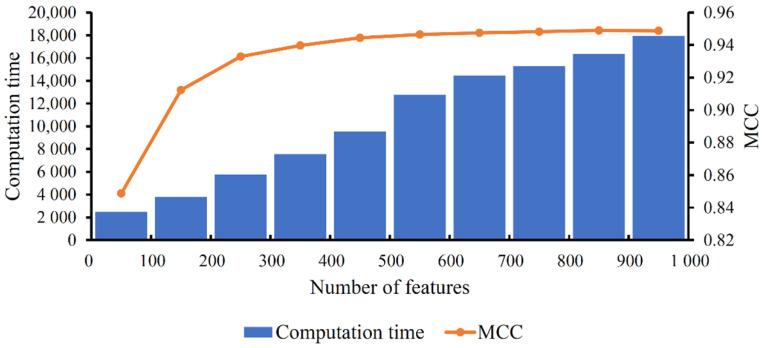
MCC and computation time of RF classifiers with different numbers of features. With the increasing feature number, the computation time follows an increasing trend, whereas MCC first follows a sharp increasing trend and then becomes stable (i.e., follows a limited increasing trend).

When constructing a tool, efficiency and accuracy are two important factors. In this study, accuracy was measured by MCC, whereas efficiency is generally assessed by computation time. To investigate the relationship of RF classifiers with different numbers of features, we counted the computation time of 10-fold cross-validation on each classifier. To exclude the influence of abnormal computation time, we grouped the computation time with the interval of 100 features and computed the average time for each group. At the same time, the average MCC in each group was also computed. These average values of MCC and computation time are summarized in Figure 5. It can be observed that the computation time always followed an increasing trend with the increase in feature number, whereas the trend of MCC was different. It first followed a sharp increasing trend and then became stable. When the feature number reached 300, the increase in MCC was quite limited. Although an increasing number of features were added, inducing an increasing amount of computation time, MCC did not obviously increase. In view of this, the feature number between 200 and 300 was a good choice to construct the tool. This was the reason why we selected the RF classifier with the top 225 features as the tool to classify cell types. The above results also indicated that the feature selection method can help us find a balance between efficiency and accuracy. We can, therefore, determine a classifier using a small number of features and a high accuracy.

### 3.5. Analysis of Features

By using the IFS method with RF, we identified a group of features that was very significant for the classification of inflammatory skin disease and healthy control cells. The optimal RF classifier can use 795 gene expression patterns as features to classify cell types with an ACC of 0.951. However, the RF classifier with top 225 features also yielded quite high performance and needed much fewer features. The 225 features used in this classifier were more important than the following 570 features used in the optimal RF classifier. Thus, we only focused on these gene features. At the same time, they also characterize inflammatory skin disease and provide potential therapeutic targets.

Among the top 10 ranked features in our results, five genes encode heterodimers that are composed of MHC class II molecules. HLA-DRA, HLA-DQA1, and HLA-DPA1 encode HLA class II alpha-chain paralogs, and HLA-DRB1 encodes an HLA class II beta-chain paralog. Those genes are highly expressed in antigen-presenting cells, such as B lymphocytes, dendritic cells, monocytes, and macrophages, which play critical roles in immune response, given that they can present a variety of antigen peptides to provide the capability to respond to a variety of pathogens [39]. MHC genes are also linked to a variety of skin diseases, such as localized scleroderma and psoriasis [40,41]. These diseases are often associated with MHC-related autoimmunity and inflammation. This finding proves the reliability of our results because our data were from normal skin and inflammatory skin disease cells, and HLA is very important for their classification.

PTPRC (ENSG00000081237), which is also known as CD45, encodes a member of the protein tyrosine phosphatase family and is important for regulating T- and B-cell antigen receptor signaling. It is a commonly used lymphocyte marker in flow cytometry because it is expressed in all nucleated hematopoietic cells [42]. CD45 deficiency can cause severe immune dysfunction and is associated with numerous diseases, such as autoimmune diseases and cancer [43,44].

The protein encoded by PERP (ENSG00000112378) is a component of intercellular desmosome junctions. It is a p63/p53 regulated gene that is essential for epithelial integrity and cell–cell adhesion [45]. PERP is highly expressed in keratinocytes, and the loss of PERP expression can weaken cell–cell adhesion at the leading edge of the wound and impairs wound repair [46].

CD74 (ENSG00000019582) is a protein-coding gene and is critical in MHC class II antigen processing. It is highly expressed in B cells and macrophages and could also be used as a biomarker [47]. The association of CD74 with skin inflammation and skin fibrosis [48,49] proves that the CD74 level could be a significant feature for distinguishing different cell types in healthy skin and inflammatory skin disease samples.

The protein encoded by TM4SF1 (ENSG00000169908) is a member of the transmembrane 4 superfamily. TM4SF1 is highly expressed in fibroblasts and endothelial cells [50,51] and is important for endothelial cell adhesion, proliferation, and migration. The expression level of the DSP gene (ENSG00000096696) is also a signature of some fibroblasts [50]. The DSP gene encodes the desmoplakin protein and acts as an anchor for intermediating filaments to desmosomal plaques. Given that DSP mutations can cause keratodermas, such as skin fragility–woolly hair syndrome, the regular expression of DSP is crucial for the normal functioning of skin cells [52].

Several genes, such as FCER1G, IDO1, and CD83, are associated with inflammatory processes. These inflammation-related genes may act as key features for distinguishing skin cells derived from healthy samples from those originating from inflammatory skin disease samples. The FCER1G gene (ENSG00000158869) encodes the Fc fragment of IgE and is involved in transmembrane signaling receptor activity and IgE binding. FCER1G is strongly upregulated in antigen-presenting cells in patients with AD, and the demethylation of FCER1G is related to the overexpression of immunoglobulin E in monocytes and dendritic cells and may be the cause of atopic dermatitis [53]. The downregulation of FCER1G in mast cells can alleviate the skin inflammatory response [54]. The protein encoded by the IDO1 gene is a heme enzyme that participates in the catabolism of tryptophan. IDO1 plays a role in a variety of physiological and pathological processes, such as immune regulation, neuropathology, and antioxidant activity. Studies have proven the importance of IDO1 in skin inflammatory disease [55]. The downregulation or inhibition of IDO1 can accelerate skin wound healing by affecting the expression of proinflammatory cytokines and chemokines [56]. IDO1 is highly expressed in skin samples from patients with psoriasis [57] and is also abnormally expressed in patients with AD. Upon viral stimulation, IDO1 is highly expressed at a significant level in the Langerhans cells of patients with AD. In addition, the IDO1 expression of plasmacytoid dendritic cells is up-regulated in patients with AD [58]. CD83 (ENSG00000112149) encodes a single-pas type I membrane protein that is a member of the immunoglobulin superfamily of receptors. The increased number of mature CD83+ dendritic cells is a distinct cellular feature of psoriatic skin. Normal skin contains only 0–5 CD83+ dendritic cells per area; this number can increase to more than 100 in active psoriatic lesions [59,60]. CD83 is highly expressed in the dendritic cells of patients with AD, and CD83+ dendritic cells can directly stimulate T-cell activation in the skin lesions of AD and psoriasis [61]. These immune-related genes can be used as significant features for distinguishing skin disease cells from healthy control cells given their importance in the occurrence and development of inflammatory skin diseases, thereby further supporting the reliability of our results.

### 3.6. Analysis of the Rules

A large number of decision rules were established on the basis of the DT model, and cells can be distinguished into 3 main groups and 114 subtypes, with an accuracy of 0.815, by using the top 805 features. Below, we discuss some classification rules to verify the reliability of our results.

The protein encoded by DMKN (ENSG00000161249) is dermokine, which is also known as epidermis-specific secreted protein SK30/SK89. It was first observed to be expressed in differentiated skin layers. In our decision rules, the expression level of DMKN in the differentiated keratinocytes of all three sets of our samples was higher than that in undifferentiated keratinocytes. This finding was consistent with the previous results showing that DMKN regulates keratinocyte growth and differentiation and that DMKN deficiency can cause skin keratinization defects in mice [62]. Moreover, DMKN has been found to be upregulated in inflammatory skin disorders, including psoriasis and AD, via a possible pathogenesis mechanism in which a high DMKN level leads to the upregulation of chemokines and cytokines and further affects the growth and differentiation of keratinocytes and the dermal recruitment of neutrophils [63]. Thus, the differential expression of DMKN can be used as a decisive criterion for reflecting the varying differentiation and pathological states of keratinocytes.

In our decision rules for the identification of T helper cells, CD3E and IL7R are required to be highly expressed in all three groups. CD3E (ENSG00000198851) can be used as a marker for T cells because it encodes the T-cell surface glycoprotein CD3 epsilon chain. It plays a vital role in T-cell development and antigen recognition, and its deficiency causes severe combined immunodeficiency [64]. Interleukin 7 receptor is the protein encoded by the IL7R gene (ENSG00000168685). It plays a critical role in lymphocyte development, and IL7R mutation may also lead to severe combined immunodeficiency [65]. A study on autoimmune disease found that IL7R is essential for the survival and expansion of pathogenic T helper types 17 [66]. Although whether IL7R has an important role in T helper cells in inflammatory skin diseases remains unclear, this result provides us with inspiration for further research. In addition, one gene was differentially expressed between patients with psoriasis and healthy controls. Our decision rules showed that T helper cells require lower TXNIP levels in patients with psoriasis than in healthy controls. TXNIP (ENSG00000265972) encodes a thioredoxin-binding protein that can regulate cellular redox signaling and protect cells from oxidative stress [67]. Studies have demonstrated that TXNIP is hypermethylated in psoriasis samples relative to in control samples, and TXNIP is downregulated in psoriatic skin [68,69]. This result may provide us with criteria for distinguishing psoriasis cells from other cells.

The PLVAP gene encodes for the plasmalemma vesicle-associated protein, which is required for the formation of the stomatal diaphragms associated with certain endothelial fenestrations and the caveolar membrane system [70]. It is a crucial component of vascular homeostasis, and a study on mutant mice found that the lack of PLVAP results in subcutaneous edema, hemorrhages, and defects in the vascular wall of subcutaneous capillaries [71]. The high PLVAP levels that usually appear in tumor endothelial cells may be related to tumor vascular proliferation and increased permeability [72]. In our results, the decision rules showed that vascular endothelial cells require relatively high PLVAP expression. Moreover, our decision rules indicated that the vascular endothelium of patients with AD also requires a relatively high expression of SOCS3. SOCS3 (ENSG00000184557) is a protein-coding gene that encodes a member of the STAT-induced STAT inhibitor family. Other studies have confirmed that SOCS3 is significantly more highly expressed in the skin of patients with AD than in the skin of healthy controls or patients with psoriasis and that the high SOCS3 levels in patients with AD may be related to T helper cells [73,74].

In our decision rules for the identification of inflammatory macrophages, the CLEC10A and CXCL8 genes are required to be highly expressed by inflammatory macrophages in inflammatory skin disease samples. The protein encoded by CXCL8 (ENSG00000169429) is interleukin-8, which is a member of the CXC chemokine family and a major mediator of the inflammatory response. Considerable data have shown that CXCL8 can be secreted by monocytes, macrophages, neutrophils, eosinophils, T lymphocytes, epithelial cells, and fibroblasts after appropriate stimulation [75]. Several studies have illustrated the important role of CXCL8 in acute and chronic inflammatory conditions and cancer [76,77]. In accordance with our decision rules, CXCL8 is highly expressed in multiple cell types in psoriasis skin samples [78]. High CXCL8 levels can activate the release of inflammatory mediators, thus leading to the inflammation and migration of neutrophils to the lesion [79,80]. CXCL8 is also upregulated in patients with AD and is involved in the inflammatory response [81,82]. The protein encoded by CLEC10A is a member of the C-type lectin/C-type lectin-like domain superfamily and is also known as macrophage lectin 2. It is most highly expressed on dendritic cells and macrophages [83]. The high CLEC10A level in our decision rules was in line with a previously reported result showing that CLEC10A is upregulated in patients with psoriasis or AD and is particularly highly expressed in macrophage and dendritic cells [84,85]. In patients with AD, drug intervention decreases CLEC10A expression and skin immune infiltration and relieves inflammation [86].

Overall, the criteria of our decision rules for different cell types are usually cell markers or are important for maintaining the function of a specific cell type (e.g., DMKN, CD3E, CLEC10A). Consistent with the inflammatory microenvironment that is widespread in inflammatory skin diseases, numerous criteria for different pathological conditions are based on the expression level of inflammation-related genes. However, the classification criteria for some cell types have not been clearly studied (e.g., IL7R, PLVAP, SOCS3, CXCL8), and our decision rules showed that their aberrant expression was associated with inflammatory skin disease. The expression levels of these genes may have an effect on the occurrence and development of diseases and may become potential therapeutic targets.

## 4. Conclusions

In this study, a computational flow with feature selection methods and classification algorithms was designed to detect the key gene features and decision rules in the single-cell expression profiles of inflammatory skin disease samples and healthy controls. These findings can uncover essential expression differences in inflammatory skin diseases and healthy controls, thereby helping us correctly diagnose such skin diseases. Furthermore, the RF classifier using fewer gene features had superior performance, which can be a useful tool to classify the cell types in inflammatory skin disease samples and healthy controls at the single-cell level. Such a classifier and the decision rules can be applied to new datasets if they are produced in the same way as the dataset investigated in this study. The reliability of our findings was verified by recent publications. Thus, this study provides new insights into future research on skin disease. Finally, the proposed computational flow is quite general in scope, suggesting it can be used to analyze expression profiles of various diseases. The codes used in this study are provided in File S1.

## Figures and Tables

**Table 1:** Performance of some key classifiers.

Classification Algorithm	Number of Features	ACC	MCC
Random forest	795	0.951	0.949
Random forest	225	0.932	0.931
Decision tree	805	0.815	0.810

## Data Availability

The data presented in this study are openly available at https://developmentcellatlas.ncl.ac.uk/datasets/hca_skin_portal (accessed on 15 January 2021).

## Supplementary Materials

The following supporting information can be downloaded at: https://www.mdpi.com/article/10.3390/life12040550/s1, Table S1: Sample sizes of each cell type in inflammatory skin disease samples and healthy controls, Table S2: Feature list including 3758 gene features obtained by using the maximum relevance criterion, Boruta feature selection, and MCFS methods. The features were sorted in descending order on the basis of the RI scores evaluated through the MCFS method. The last three features were removed because their RI score was 0, Table S3: Performance of the DT and RF classifiers with different numbers of features, mainly including the MCC of 114 classes and overall accuracy, Table S4: Confusion matrices of some key classifiers, Table S5: Top 1000 classification rules extracted by the optimal DT classifier, File S1: Codes of this study.
