# Supplementary material for: Identification of Cell Markers and Their Expression Patterns in Skin Based on Single-Cell RNA-Sequencing Profiles
Source: Life (Basel). 2022 Apr 7;12(4):550. doi: 10.3390/life12040550 (PMC9025372; doi:10.3390/life12040550)
Supplement: Supplementary file 1 [file life-12-00550-s001.zip › File S1.pdf]

File S1. Codes of this study.

Guide for using codes

Step1. Download the single-cell expression profiles at https://developmentcellatlas.ncl.ac.uk/datasets/hca_skin_portal.

Step2. The profiles were analyzed by mRMR program (http://penglab.janelia.org/proj/mRMR/). Features with MI no less than 0.001 were extracted.

Step3. The profiles with remaining features were analyzed by Boruta program (https://github.com/scikit-learn-contrib/boruta_py). Important features were further extracted.

Step4. The profiles with remaining features were finally analzyed by MCFS program (http://www.ipipan.eu/staff/m.draminski/mcfs.html). A feature list was obtained with the decreasing order of RI values.

Step5. Based on such list, several data files with step five were produced with the following command:

python exportDimsCSV.py -d datatsets/original_file -i datatsets/feature_order_original_file -b 5 -e 2778 -s 5

Step6. For each produced data file, run the program of random forest and decision tree with the following commands:

- python sk-rf-kfold-smote-run.py --datapath input_file -t 100 -d 1000 -e 100 -r 88 -k 10 -n 2 > output_file
- python sk-dt-smote-kfold-run.py -k 10 --datapath input_file > output_file

Step7. Count the results in the obtained result files.

Codes in sk-rf-kfold-smote-run.py

```
import time
from argparse import ArgumentDefaultsHelpFormatter, ArgumentParser

__author__ = 'Min'


if __name__ == "__main__":
    start_time = time.time()
    parser = ArgumentParser(description="A random forest classifier based on scikit-learn with SMOTE apply it to classify bio datasets.",
                            formatter_class=ArgumentDefaultsHelpFormatter)

    parser.add_argument("-t", "--ntrees", type=int,
                        help="The number of trees in the forest.", default=100)
    parser.add_argument("-d", "--mdepth", type=int,
                        help="The maximum depth of the tree. If None, then nodes are expanded until all leaves are pure or until all leaves contain less than min_samples_split samples.", default=None)
    parser.add_argument("-e", "--epochs", type=int,
                        help="Number of training epochs.", default=100)
    parser.add_argument("-k", "--kfolds", type=int,
                        help="Number of folds. Must be at least 2.", default=10)
    parser.add_argument("-r", "--randomseed", type=int,
                        help="pseudo-random number generator state used for shuffling.", default=0)
    parser.add_argument("-n", "--kneighbors", type=int,
                        help="number of nearest neighbours to used to construct synthetic samples.", default=3)
    parser.add_argument("--datapath", type=str,
                        help="The path of dataset.", required=True)

    args = parser.parse_args()
    # logdir_base = os.getcwd()


    import numpy as np
    import pandas as pd
    from sklearn.ensemble import RandomForestClassifier
    from sklearn.model_selection import cross_val_predict
    from imblearn.over_sampling import SMOTE
    from lgb.utils import model_evaluation, bi_model_evaluation
```

```
df = pd.read_csv(args.datapath)

X = df.iloc[:, 1:].values
y = df.iloc[:, 0].values - 1
# f_names = df.columns[1:].values

print("\nDataset shape: ", X.shape, " Number of features: ", X.shape[1])
num_categories = np.unique(y).size
sum_y = np.asarray(np.unique(y.astype(int), return_counts=True))
df_sum_y = pd.DataFrame(sum_y.T, columns=['Class', 'Sum'], index=None)
print('\n', df_sum_y)


sm = SMOTE(k_neighbors=args.kneighbors, random_state=args.randomseed, n_jobs=-1)
x_resampled, y_resampled = sm.fit_sample(X, y)
# after over sampleing
np_resampled_y = np.asarray(np.unique(y_resampled, return_counts=True))
df_resampled_y = pd.DataFrame(np_resampled_y.T, columns=['Class', 'Sum'])
print("\nNumber of samples after over sampleing:\n{0}\n".format(
    df_resampled_y))


clf = RandomForestClassifier(
    n_jobs=-1,                  n_estimators=args.ntrees,                   max_depth=args.mdepth,
random_state=args.randomseed)
print("\nClassifier parameters:")
print(clf.get_params())
print("\nSMOTE parameters:")
print(sm.get_params())
print("\n")

y_pred_resampled = cross_val_predict(
    clf, x_resampled, y_resampled, cv=args.kfolds, n_jobs=-1, verbose=1)

y_pred = y_pred_resampled[0:X.shape[0]]


if(num_categories > 2):
    model_evaluation(num_categories, y, y_pred)
else:
    bi_model_evaluation(y, y_pred)
end_time = time.time()
print("\n[Finished in: {0:.6f} mins = {1:.6f} seconds]".format(
```

((end_time - start_time) / 60), (end_time - start_time)))

Codes in sk-dt-smote-kfold-run.py

```
import time
from argparse import ArgumentParser, ArgumentDefaultsHelpFormatter

__author__ = 'Min'


if __name__ == "__main__":
    start_time = time.time()
    parser = ArgumentParser(description="A decision tree classifier with SMOTE based on scikit-
learn apply it to classify bio datasets.",
                            formatter_class=ArgumentDefaultsHelpFormatter)
    parser.add_argument("-k", "--kfolds", type=int,
                        help="Number of folds. Must be at least 2.", default=10)
    parser.add_argument("-r", "--randomseed", type=int,
                        help="pseudo-random number generator state used for shuffling.",
default=0)
    parser.add_argument("--datapath", type=str,
                        help="The path of dataset.", required=True)

    args = parser.parse_args()


    import numpy as np
    import pandas as pd
    from sklearn.tree import DecisionTreeClassifier
    from sklearn.model_selection import KFold
    from sklearn.metrics import accuracy_score
    from imblearn.over_sampling import SMOTE
    from utils import model_evaluation, bi_model_evaluation


    df = pd.read_csv(args.datapath)

    X = df.iloc[:, 1:].values
    y = df.iloc[:, 0].values - 1
    f_names = df.columns[1:].values

    print("\nDataset shape: ", X.shape, " Number of features: ", X.shape[1])
    num_categories = np.unique(y).size
    sum_y = np.asarray(np.unique(y.astype(int), return_counts=True))
    df_sum_y = pd.DataFrame(sum_y.T, columns=['Class', 'Sum'], index=None)
    print('\n', df_sum_y)
```

```
    sm = SMOTE(k_neighbors=2)
    x_resampled, y_resampled = sm.fit_sample(X, y)
    # after over sampleing
    np_resampled_y = np.asarray(np.unique(y_resampled.astype(int), return_counts=True))
    df_resampled_y = pd.DataFrame(np_resampled_y.T, columns=['Class', 'Sum'])
    print("\nNumber of samples after over sampleing:\n{0}".format(df_resampled_y))


    clf = DecisionTreeClassifier(random_state=args.randomseed)
    print("\nClassifier parameters:")
    print(clf.get_params())

    rs = KFold(n_splits=args.kfolds, shuffle=True, random_state=args.randomseed)

    resampled_index_set = rs.split(y_resampled)
    k_fold_step = 1

    test_cache = pred_cache = np.array([], dtype=np.int)

    for train_index, test_index in resampled_index_set:
        print("\nFold:", k_fold_step)
        clf.fit(x_resampled[train_index], y_resampled[train_index])

        real_test_index = test_index[test_index < X.shape[0]]
        batch_test_x = x_resampled[real_test_index]
        batch_test_y = y_resampled[real_test_index]
        batch_test_size = len(real_test_index)

        y_pred = clf.predict(batch_test_x)
        # ACC
        accTest = accuracy_score(batch_test_y, y_pred)
        print("\nFold:", k_fold_step, "Test Accuracy:",
              "{:.6f}".format(accTest), "Test Size:", batch_test_size)

        #
        test_cache = np.concatenate((test_cache, batch_test_y))
        pred_cache = np.concatenate((pred_cache, y_pred))

print("\n======================================================================
==========")

        k_fold_step += 1
```

```
if(num_categories > 2):
	model_evaluation(num_categories, test_cache, pred_cache)
else:
	bi_model_evaluation(test_cache, pred_cache)
end_time = time.time()    #
print("\n[Finished in: {0:.6f} mins = {1:.6f} seconds]".format(
	((end_time - start_time) / 60), (end_time - start_time)))
```

Codes in exportDimsCSV.py

```
import time
import numpy as np
import pandas as pd
from argparse import ArgumentParser, ArgumentDefaultsHelpFormatter

__author__ = 'Min'
if __name__ == "__main__":
    start_time = time.time()
    parser = ArgumentParser(description="This program is used to export a custom dims feature matrix.", formatter_class=ArgumentDefaultsHelpFormatter)
    parser.add_argument("-d", "--datapath", type=str, help="The path of dataset.", required=True)
    parser.add_argument("-i", "--idxpath", type=str, help="The path of indexes file.", required=True)
    parser.add_argument("-b", "--start", type=int, help="An integer number specifying at which position to start. Default is 0", required=True, default=0)
    parser.add_argument("-s", "--step", type=int, help="An integer number specifying the incrementation. Default is 1", default=1)
    parser.add_argument("-e", "--stop", type=int, help="An integer number specifying at which position to endt.", default=10)
    args = parser.parse_args()

    df_features = pd.read_csv(args.datapath, encoding='utf8')
    df_idxs = pd.read_table(args.idxpath, header=None)

    print("\nThe shape of features matrix: ", df_features.shape)
    print("\nThe shape of indexes file: ", df_idxs.shape)
    print("\nStart:{0}, Stop:{1}, Step:{2}".format(args.start, args.stop, args.step))
    print("\nStart exporting custom features matrix...")


    for x in range(args.start, args.stop+1, args.step):
        if x == 0:
            pass
        else:
            new_features = df_features.iloc[:, np.insert(df_idxs.loc[:(x-1)].values.T[0], 0, 0)]
            new_features.to_csv('{0}.csv'.format(x), index=None)
            # print(df_features.iloc[:, np.insert(df_idxs.loc[:(x-1)].values.T[0], 0, 0)])

    end_time = time.time()
    print("\n[Finished in: {0:.6f} mins = {1:.6f} seconds]".format(((end_time - start_time) / 60), (end_time - start_time)))
```
